# A Dual Attention Encoding Network Using Gradient Profile Loss for Oil Spill Detection Based on SAR Images

**DOI:** 10.3390/e24101453

**Published:** 2022-10-12

**Authors:** Jiding Zhai, Chunxiao Mu, Yongchao Hou, Jianping Wang, Yingjie Wang, Haokun Chi

**Affiliations:** 1 School of Computer and Control Engineering, Yantai University, Yantai 264005, China; 2 Shandong Marine Resources and Environment Research Institute, Yantai 264006, China

**Keywords:** oil spill, SAR image, deep learning, attention module, gradient profile loss

## Abstract

Marine oil spills due to ship collisions or operational errors have caused tremendous damage to the marine environment. In order to better monitor the marine environment on a daily basis and reduce the damage and harm caused by oil pollution, we use marine image information acquired by synthetic aperture radar (SAR) and combine it with image segmentation techniques in deep learning to monitor oil spills. However, it is a significant challenge to accurately distinguish oil spill areas in original SAR images, which are characterized by high noise, blurred boundaries, and uneven intensity. Hence, we propose a dual attention encoding network (DAENet) using an encoder–decoder U-shaped architecture for identifying oil spill areas. In the encoding phase, we use the dual attention module to adaptively integrate local features with their global dependencies, thus improving the fusion feature maps of different scales. Moreover, a gradient profile (GP) loss function is used to improve the recognition accuracy of the oil spill areas’ boundary lines in the DAENet. We used the Deep-SAR oil spill (SOS) dataset with manual annotation for training, testing, and evaluation of the network, and we established a dataset containing original data from GaoFen-3 for network testing and performance evaluation. The results show that DAENet has the highest mIoU of 86.1% and the highest F1-score of 90.2% in the SOS dataset, and it has the highest mIoU of 92.3% and the highest F1-score of 95.1% in the GaoFen-3 dataset. The method proposed in this paper not only improves the detection and identification accuracy of the original SOS dataset, but also provides a more feasible and effective method for marine oil spill monitoring.

## 1. Introduction

With the rapid development of the marine industry and oil extraction projects, the growth of marine oil spills has caused tremendous damage to the marine ecological environment [1]. Early detection and identification of oil spills in terms of oil distribution, volume spilled, and oil film thickness can significantly facilitate decision making for effective cleanup programs. Therefore, timely and accurate information on oil slicks’ location and area is important to enable emergency responses to oil spills [2,3].

Traditional remote sensing technology has the advantages of wide monitoring range and high efficiency of information collection, so it is more and more widely used in the field of marine environment monitoring. Compared with other remote sensing technologies, synthetic aperture radar (SAR), as an active remote sensing technology, has the characteristics of all-time, all-weather, and strong penetration ability, and it has become an important technology for oil spill monitoring. SAR image information is usually visualized in the form of grayscale, and by analyzing the distribution of grayscale values in a local area, the surface or sea surface information of the area can be obtained. Since the oil film suppresses the short-time gravity capillary waves of the seawater surface and reduces the backscattered signal received by SAR, the oil spill area on the image usually has a higher gray value. Therefore, the oil spill area can be quickly and accurately identified by capturing the dark area in the SAR image [4,5]. At present, the methods to extract the oil spill region in SAR images by image segmentation include [6]: thresholding and the derived adaptive thresholding, edge detection, machine learning, etc. Li et al. [7] proposed a SAR image oil spill detection algorithm based on maximum entropy threshold segmentation that uses a sliding window approach to select the best segmentation threshold based on maximum entropy inside the window. Jing et al. [8] proposed a robust active contour edge detection algorithm based on a local Gaussian statistical model for oil slick remote sensing images. Oscar et al. [9] proposed a texture-classifying neural network algorithm (TCNNA) that uses information such as texture features in SAR images, wind speed in the target area, and radar incidence angle as input data for oil spill monitoring using a back propagation (BP) neural network. SAR images have high noise speckle, resulting in the gray value of the target region usually changing sharply, and traditional image segmentation algorithms are extremely vulnerable to this noise. Traditional machine learning methods are support vector machines [10], random forests [11], and BP neural networks [12]. These methods require manually designed rules or more matching information to obtain more accurate prediction results, and matching information is very difficult to obtain. For example, the above TCNNA model requires the design of texture feature calculation rules, as well as wind force, incidence angle, and other information matching the image information.

Deep learning methods have the advantages of high learning ability, high generalization ability, and good adaptability [13,14,15,16,17,18,19,20,21,22,23,24]. So far, there are many deep learning models used for image segmentation, such as the U-Net [13], which was first applied in the field of medical images [14], and the derived U-Net+ and U-Net++ models. They use a U-shaped encoder–decoder structure for multiscale feature extraction, combine low-resolution and high-resolution information, and are fully applicable to medical image segmentation, which greatly improves image segmentation accuracy. D-Linknet [15] also adopts the U-shaped encoder and decoder structure, and it adds the dilated convolution operation to the network, using Resnet34 pretrained on the Imagenet data set as its encoder. This model is very successful in the field of satellite image road extraction. DeepLabv3+ is the fourth-generation model of the DeepLab series proposed by Google, and its main body is with null convolution DCNN and ASPP; DCNN can adopt common classification networks such as ResNet, VGG, etc. Compared with the previous-generation model, DeepLabv3+ [16] introduces a decoder module to further integrate the underlying and high-level features and improve the accuracy of the segmentation boundary, and the model also has wide application in the field of semantic segmentation. The use of a deep learning model to extract the oil spill areas in SAR oil spill images can well solve the limitations of traditional methods. It does not require too much manual operation, has better versatility, and only needs image information as data input to obtain high detection accuracy. Krestenitis et al. [17] found that the DeepLabv3+ model achieved the best overall performance in oil spill detection by comparing different deep learning semantic submodels. Fan et al. [18] extracted the high-frequency components in SAR images by a threshold segmentation method; extracted the high-frequency components and the high-dimensional features of the original image by a convolution operation, then fused them separately; and constructed a feature merged network for oil spill detection using SAR images. Li et al. [19] proposed a multiscale conditional adversarial network for oil spill detection based on small data training, which enhances the representativeness of the model by generating data through adversarial training and captures global and local oil spill features using a multiscale architecture, then identifies the oil spill area. However, to accurately detect and identify the oil spill areas in SAR images, it is necessary to overcome the difficulties associated with a large and diverse amount of data for training to establish the deep learning models.

In addition, the shape of oil spill areas in SAR images is complex, and the boundary is fuzzy. Only by capturing the correlation between local features and global information can we obtain more accurate segmentation results. The convolution operation of a traditional CNN cannot capture the local features and the correlation with global features [25], and the generated local features may lead to misclassification of objects and contents [26,27,28,29,30,31]; however, the attention mechanism can effectively address this problem. Initially, a number of approaches emerged in natural language processing research to extract semantic associations [32,33,34]. Ashish et al. [26] first proposed a self-attention module to extract global dependencies of input information and applied it to natural language processing. They describe the attentional computation function as the operation of mapping two sets of associated vectors to an output vector, which are called queries and keys. The output vector is obtained by weighting the degree of association of queries and keys. Zhang et al. [27] introduced a self-attentive module in generative adversarial networks for image generation work. Fu et al. [28] introduced a dual self-attention mechanism in the semantic segmentation task, where the location attention module is used to capture global information and the channel attention block is used to extract dependencies between channels.

In this study, we propose a dual attention encoding network (DAENet) using an encoder–decoder U-shaped architecture. A dual attention encoding module with a Position Attention Module (PAM) is used to capture global information and obtain pixel-level relationships at different locations within the same channel; CAM is used to capture pixel-level relationships at the same locations within different channels. Then, the gradient profile (GP) loss is used as an additional loss function to sharpen the edge part of the target region in SAR images and improve the edge region accuracy. Based on experiments training and testing the network using the Deep-SAR Oil Spill (SOS) dataset and GaoFen-3 self-made dataset, the practical use of the network is demonstrated, enabling accurate recognition of oil spill areas in SAR images.

## 2. Materials and Methods

### 2.1. Dataset

#### 2.1.1. SOS Dataset

**Figure 1:**
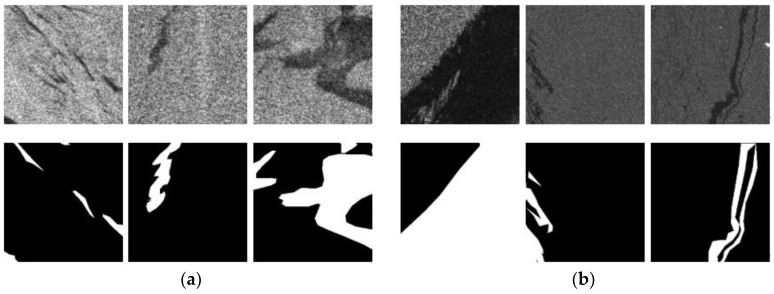
Selected image data from the Deep-SAR Oil Spill (SOS) dataset (the white area is the oil spill area, the black area is the background area): (**a**) Example data from PALSAR; (**b**) Example data from Sentinel-1A.

In this paper, the Deep-SAR Oil Spill (SOS) dataset proposed by Zhu et al. [21] was used to train and test the model. It consists of two parts: (1) 14 PALSAR images of the Gulf of Mexico region with pixel spacing of 12.5 m and HH polarization as the polarization mode; and (2) 7 Sentinel 1A images of the Persian Gulf region with a spatial resolution of 5 m × 20 m and VV polarization as the polarization mode. Subsequently, the original images were enhanced by image augmentation techniques (cropping, rotating, and adding noise) to enhance the diversity of the data set, and 21 original images were divided into 6456 416 × 416 pixel oil leakage images. Based on this, data labels were made by manual labeling, and expert sampling was performed. The final dataset consisted of 8070 256 × 256 pixel images of the oil spill with labels. The dataset and test set were divided in the ratio of 8:2. The training set included a total of 3101 images from PALSAR and 3354 images from Sentinel-1. The test set included a total of 776 images from PALSAR and 839 images from wave Sentinel-1. Some sample data from the Deep SAR Oil Spill (SOS) dataset are shown in Figure 1 [21].

#### 2.1.2. GaoFen-3 Dataset

**Figure 2:**
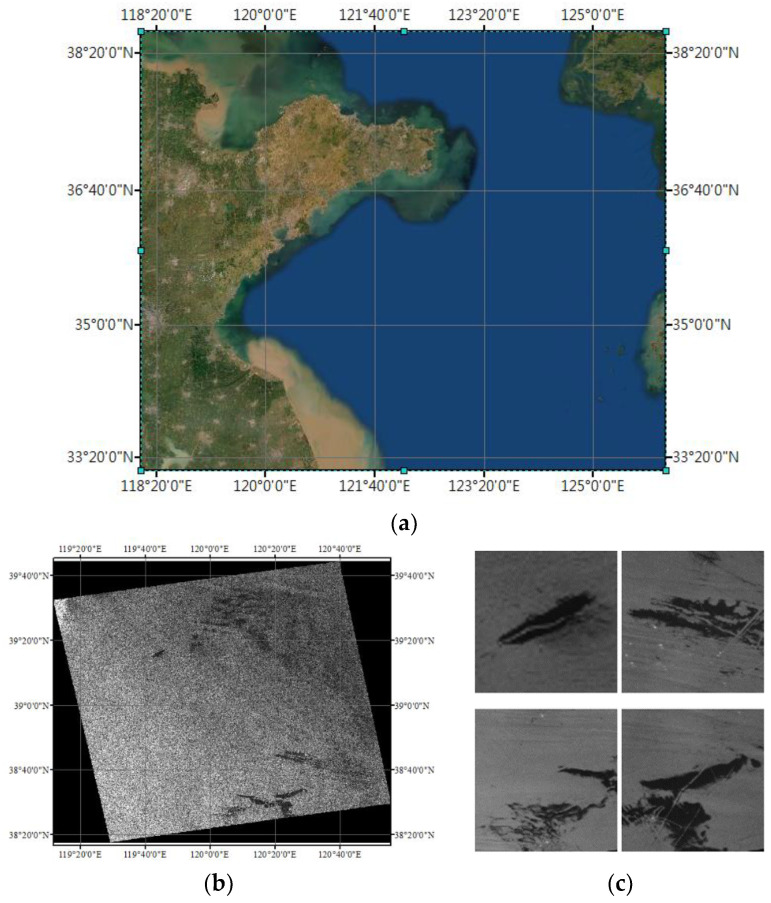
GaoFen-3 satellite image data: (**a**) SAR image target area; (**b**) GaoFen-3 raw image data; (**c**) A subset of our selected oil spill area.

In order to further test the performance of the model, we acquired data from Gaofen-3 satellite images for validation experiments. The acquired image data are shown in Figure 2.

**Figure 3:**
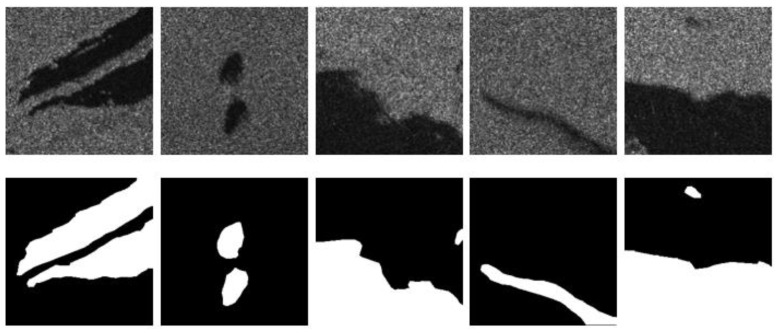
Part of the image data from the Gaofen-3 satellite data set (the white area is the oil spill area, the black area is the background area).

The data were collected on 9 May 2021, in the offshore area of Qingdao. The width covered by the images is 130 km, and their spatial resolution is 25 m. The polarization mode is VV. In the process of making the dataset, the original images were first divided into several data subsets containing oil spill areas, and then 708 oil spill images of 256 × 256 pixels in size were obtained by cropping and rotating using the technique of data enhancement. It is worth noting that the operation of changing the gray level of the image or adding noise was not carried out in the process of data enhancement, while the rotation operation was used to randomly rotate the picture by 90°, 180°, or 270°. Then, the sample labels were made by a manual annotation method for the subsequent testing work. Some of the image data from this dataset are shown in Figure 3.

### 2.2. Dual Attention Encoding Network (DAENet)

#### 2.2.1. Encoder–Decoder Architecture

**Figure 4:**
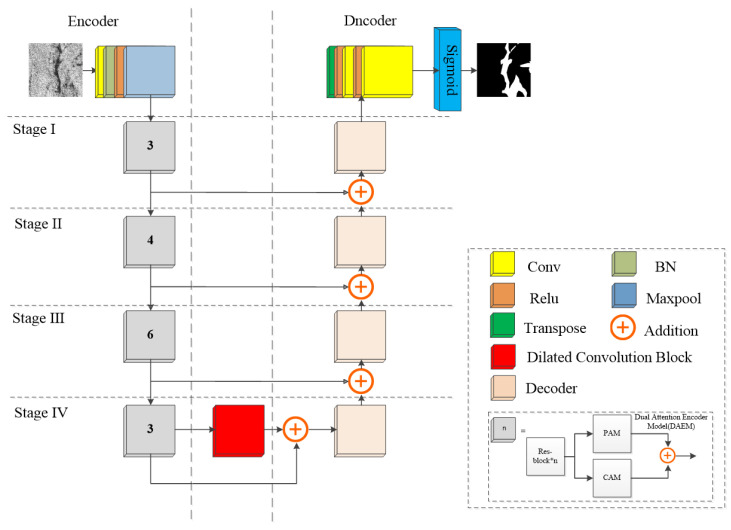
Overview of the proposed DAENet.

DAENet is an oil spill detection network based on SAR images. It uses an encoder–decoder U-shaped architecture, as shown in Figure 4. This is an end-to-end image segmentation technology architecture. In the encoding process, feature extraction is performed at different scales by multiple encoder blocks. Then, the decoder fuses the feature maps of different scales and performs an upsampling operation to obtain the detection results.

In DAENet, the basic features are first extracted from the input image using the first stage of ResNet [35], a convolutional layer with a kernel size of 7 × 7, and a step size of 2. The extracted preliminary features are then processed using a batch normalization (BN) layer, a Relu layer, and a Maxpool layer, and the results are provided to the dual attention encoder (DAE). The BN layer is used to prevent the gradient from disappearing or exploding during the training process, while speeding up the training. The Relu layer is an excitation function to add nonlinear factors. The Maxpool layer can reduce the parameters and calculation while retaining the main features, so as to prevent overfitting. The DAE consists of four dual attention encoding modules (DAEMs) stacked on top of each other. In each stage of the DAEMs, the initial features are first extracted by the residual module (Res-block), and then the extracted initial features are sent to the position attention module (PAM) and the channel attention module (CAM). The processing results of the PAM and CAM are fused to construct a multiscale feature map, which is passed to the encoding module in the next stage and to the decoding module in the same stage. The numbers labeled by the DAEMs at each stage in Figure 4 represent the number of residual modules, such that the structure is an encoder structure with ResNet-34 as the backbone. The parameters of the ResNet-34 structure in the encoder use the values pretrained on ImageNet [36] as the initial parameters, which results in better performance and can improve the convergence speed of the model during training.

The original image of size 256 × 256 is processed by DAE to obtain a feature map of size 8 × 8. DAENet connects a dilated convolution block after the encoder, which expands the receptive field while preserving spatial information. It consists of a stack of 4 dilated convolution layers, and the processing result of each dilated convolution layer is involved in the final matrix addition operation. The dilation rates of the stacked dilated convolution layers are {1,2,4,8}, and in order to obtain an output feature map of the same size as the input feature map, the padding is {1,2,4,8}, and the stride sizes are all 1. According to Equation (1), the receptive field size of each layer is calculated as {3,7,15,31}:

(1) K=D×F+(D−1)

where K is the receptive field size, D is the convolution kernel size, and F is the dilation rate.

The feature map output from the dilated convolution block is fed to the decoder. The decoder consists of four decoder blocks, and the sum of the feature matrix coming from the lower stage and the feature matrix coming from the corresponding stage of the decoder is used as the input data of the decoder block at that stage. The data in the decoder block first pass through a convolutional layer with a kernel size of 1 × 1, and the output channel is 1/2 of the input channel; then, there is a transposed-conv layer with a kernel size of 3 × 3 and a step size of 2; finally, a convolutional layer with a kernel size of 1 × 1 is input. The feature map is upsampled to twice the original size. Each convolutional layer in the process is followed by batch normalization and Relu operations. After four decoding blocks, the feature map is fed to the last transposition-conv layer, with a kernel size of 4, an output channel of 1/2 the input channel, a step size of 2, and padding = 1. At this point, the size of the feature map is restored to the size of the original image. Then, after two Relu layers and two conv layers, a feature map of size 1 × 256 × 256 is finally obtained. The feature map after Sigmoid processing is the prediction result. It is worth noting that the step size and kernel selection of the DAENet structure were determined by performing several experiments and examining some classical deep learning models [13,15,16].

#### 2.2.2. Dual Attention Encoding Module (DAEM)

**Figure 5:**
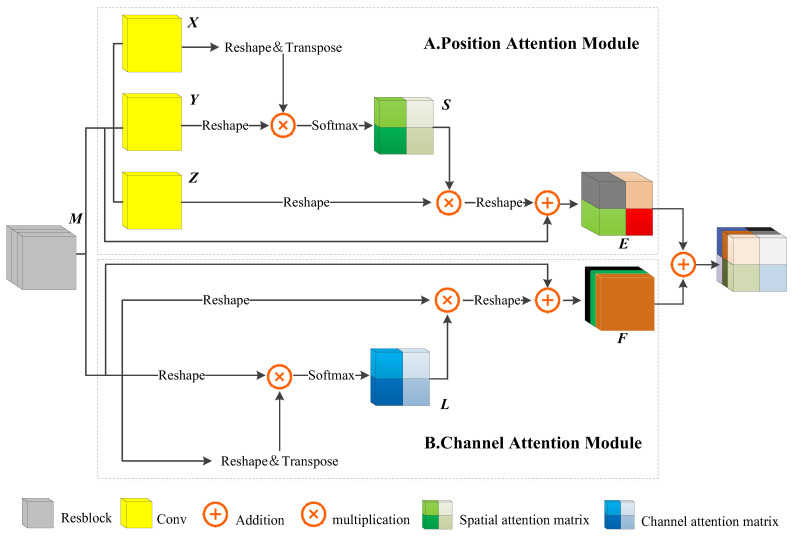
Overall view of the DAEM.

To improve the ability of the model to capture global information, we use the dual attention encoding module (DAEM) as the encoding module of DAENet, which is composed of a Resblock, a position attention module (PAM), and a channel attention module (CAM), as shown in Figure 5.

The PAM is shown in Figure 5A. The feature maps of $M\in R^{C\times H\times W}$ passed by the Res-block module are fed into the three convolution layers, and the three feature maps are obtained as $\{X,Y\}\in R^{e\times H\times W}$, $Z\in R^{C\times H\times W}$, where $e=\frac{C}{n}$. Then, X and Y are reshaped into $R^{C\times N}$, N=H×W. The transpose of X is obtained and a matrix multiplication operation is performed with Y; position attention feature map $S\in R^{N\times N}$ is then obtained after the Softmax layer:

(2) $$S_{ji}=\frac{\exp(X_{i}\times Y_{j})}{\sum_{i=1}^{N}\exp(X_{i}\times Y_{j})}$$

In Equation (2), $S_{ji}$ indicates the degree of association between the $i_{th}$ pixel value and the $j_{th}$ pixel value.

While performing the above operation, $X\in R^{C\times H\times W}$ is reshaped into $R^{C\times N}$, and then the result is reshaped into $R^{C\times H\times W}$ after performing a matrix multiplication operation between S and Z. Finally, the result of the above operation is multiplied by the scale parameter α, and a matrix addition operation is performed with M to obtain the final output $E\in R^{C\times H\times W}$:

(3) $$E_{j}=\alpha \sum_{i=1}^{N}(S_{ji}Z_{i})+M_{j}$$

In Equation (3), α is initialized to 0, and more weight is gradually allocated during the learning process. This module models the pixel-level relationships at different locations within the same channel.

Figure 5B shows the structure of the CAM. The feature map of $M\in R^{C\times H\times W}$ passed from the Res-block module is reshaped into $R^{C\times N}$, the matrix is matrix multiplied with the transpose of this matrix, and then the channel attention map $L\in R^{N\times N}$ is obtained after the Softmax layer:

(4) $$L_{ji}=\frac{\exp(M_{i}\times M_{j})}{\sum_{i=1}^{N}\exp(M_{i}\times M_{j})}$$

In Equation (4), $L_{ji}$ indicates the degree of association between the $i_{th}$ channel and the $j_{th}$ channel.

While performing the above operation, we multiply M by its transpose and then reshape the result to $R^{C\times H\times W}$. The result is then multiplied by the scale parameter β, and a matrix addition operation is performed with M to obtain the final output $F\in R^{C\times H\times W}$:

(5) $$F_{j}=\beta \sum_{i=1}^{N}(L_{ji}M_{i})+M_{j}$$

In Equation (5), β is initialized to 0, and more weight is gradually allocated during the learning process. This module constructs a model of the interdependencies between different channels.

By describing the PAM and CAM in detail, we find that they can model the ability to extract relational features between different pixels in both spatial and channel dimensions. In the literature [37,38] and in the analysis of the experimental results in Section 3, we find that this ability is effective in improving the accuracy and robustness of the models.

### 2.3. Gradient Profile Loss

In a binary classification problem, the goal of the classification is often composed of two parts: positive and negative. The output of the deep learning network after the Sigmoid activation function takes values within [0, 1]; the label values are composed of a positive, representing 0, and a negative, representing 1. The loss function is a metric function used to calculate the similarity between label values and predicted values. The training of deep learning networks usually requires the choice of one or more loss functions. When training a binary classifier, binary cross-entropy (BCE) and dice loss [39] are often used, shown in Equation (6):

(6) $$\begin{array}{l} L_{BCE}=-\frac{1}{N}\sum \left(target\times \ln(pt)+(1-target)\times \ln(1-pt)\right)\\ L_{Dice}=1-\frac{2\sum target\times pt}{\sum \left(target^{2}+pt^{2}\right)} \end{array}$$

where target is the actual label value and pt is the predicted value.

The boundary line of the oil spill area in SAR images is usually blurred, which is one of the reasons for the reduced accuracy of oil spill monitoring. To obtain more accurate detection results, we improve the ability of the model to identify the target boundary lines by introducing gradient profile (GP) loss [19,40,41]. It is worth noting that both BCE loss and dice loss calculate the loss by computing the difference between individual pixels, while the GP loss function is calculated by considering each row and column within a single channel of the image as a vector, and then calculating the cosine similarity between the real image and the target image in that vector space by computing Equation (7):

(7) $$L(x,X)=\sum_{c}\left(\frac{1}{H}\mathrm{trace}(x_{c}\times X_{c}^{T})+\frac{1}{W}\mathrm{trace}(x_{c}^{T}\times X_{c})\right)$$

where x denotes the labeled image output from the model, X denotes the actual labeled image mask, c denotes each image channel, ${\left(\cdot \right)}^{T}$ is the transpose of the matrix, and trace(⋅) is the L2 normalization. The first term in the formula is the calculation of row profile similarity between (x,X) of images of size H×W, and the second term is the calculation of column profile similarity. The gradient profile loss (GP loss) is calculated by Equation (8):

(8) $$L_{GP}=-L(\nabla x,\nabla X)$$

Due to different computational methods, different loss functions have different advantages and limitations, and sometimes a combination of different loss functions will yield better performance. Therefore, we combined BCE loss, dice loss and GP loss by constructing a joint loss function (Equation (9)).

(9) $$L=L_{BCE}+L_{Dice}+L_{GP}$$

## 3. Results and Discussion

### 3.1. Evaluation Criteria

We used the mIoU and F1-score as evaluation metrics, calculated as shown in Equation (10). True Positive (TP) indicates a positive category that was correctly determined to be positive. False Positive (FP) indicates a positive category that was falsely judged as negative. False negative (FN) indicates a negative category falsely judged to be positive. True negative (TN) indicates a negative category correctly judged as negative.

(10) $$\begin{array}{l} mIoU=\frac{1}{k+1}\sum_{i=0}^{k}\frac{TP}{TP+FP+FN}\\ Precision=\frac{TP}{TP+FP}\\ Recall=\frac{TP}{TP+FN}\\ F1\text{-}sorce=\frac{2\times Precision\times Recall}{Precision+Recall} \end{array}$$

### 3.2. Comparison of DAENet with Other Technologies

We trained the U-Net, D-LinkNet, DeepLabv3, and DAENet networks separately using the training set from the SOS dataset. Among them, the D-LinkNet and DeepLabv3 model both use RestNet34 as a backbone. All models were trained for 120 epochs. This was to ensure that all models (including comparison models) could achieve the best results. PAM compresses the channels to 1/n of the original channels in the process of generating spatially noticed feature maps, and we set n to 4, 8, and 16 for experiments, denoted as DAENet_4, DAENet_8, and DAENet_16, respectively. All used the joint loss function [17] of BCE loss, dice loss, and SSIM loss [42] as the loss function, i.e., Equation (12).

(11) $$L_{SSIM}=1-\frac{\left(2\mu_{x}\mu_{y}+C_{1})(2\sigma_{xy}+C_{2}\right)}{\left(\mu_{x}^{2}+\mu_{y}^{2}+C_{1}\right)\left(\sigma_{x}^{2}+\sigma_{y}^{2}+C_{2}\right)}$$

(12) $$L=L_{BCE}+L_{Dice}+L_{SSIM}$$

In Equation (12), x and y denote the labeled and predicted values, respectively; $\mu_{\left(\cdot \right)}$ and $\sigma_{\left(\cdot \right)}$ denote the mean and standard deviation, respectively; $\sigma_{xy}$ denotes their covariance; and both $C_{1}$ and $C_{2}$ are very small numbers to avoid the case of a zero denominator—we defined them as e-5.

After 120 epochs of training, each model was tested separately using the test set in SOS, and the test results are shown in Table 1. In the PALSAR test set, DAENet_16 obtained the highest mIoU and F1-score, with 84.1% and 85.1%, respectively; this was a 1.2% improvement in both metrics compared to the highest-scoring comparison model, DeepLabv3. DAENet_4 and DAENet_8 scored better than the DeepLabv3 model in both the mIoU and F1-score metrics. In the Sentinel-1 test set, DAENet_8 obtained the highest mIoU and F1-score, with 85.3% and 89.7%, respectively. The mIoU metric improved by 1.5% compared to the highest mIoU among the comparison models (for DeepLabv3), and the F1-score metric improved by 1.5% compared to the highest F1-score among the comparison models (for U-Net). DAENet_4 and DAENet_16 scored better than the comparison models in both the mIoU and F1-score metrics.

### 3.3. Ablation Study for Different Loss Functions

**Figure 6:**
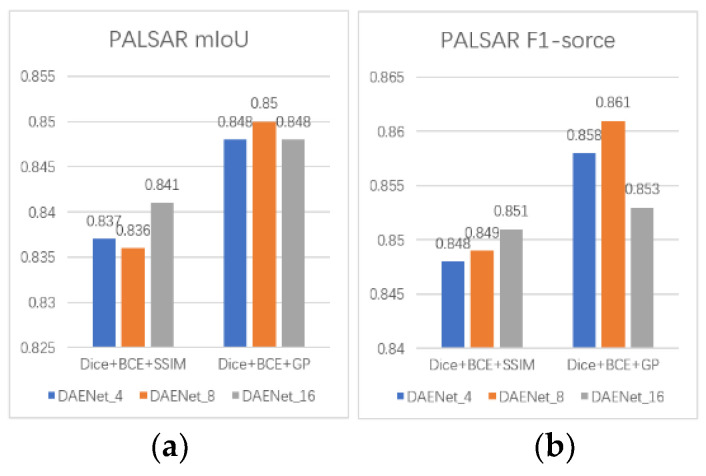

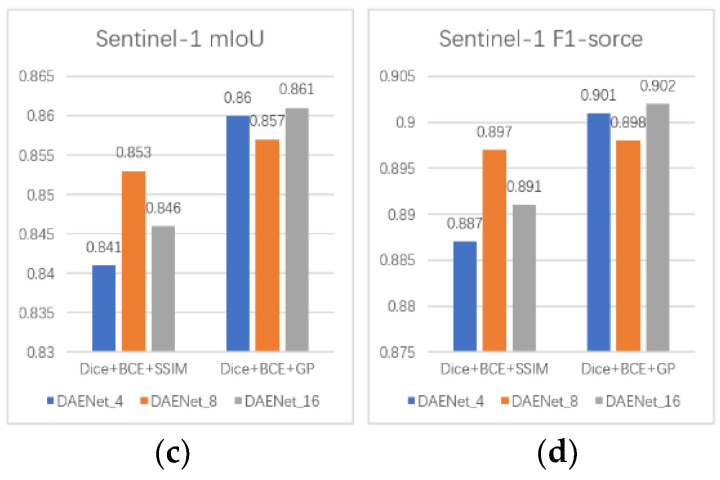
Using different loss functions in DAENet: (**a**) mIoU on the PALSAR test set; (**b**) F1-score on the PALSAR data set; (**c**) mIoU on the Sentinel-1 test set; (**d**) F1-score on the Sentinel-1 test set.

To obtain a more accurate boundary line of the oil spill area, we conducted ablation experiments on DAENet_4, DAENet_8, and DAENet_16 using Equations (9) and (12) as loss functions. The rest of the experimental procedure was the same as that in the previous section. The mIoU and F1-scores of the model in both the PALSAR and Sentinel-1 test sets are shown in Figure 6.

Both the mIoU and F1-score of the DAENet were improved after using Equation (9) as the new loss function. In the PALSAR test set, the mIoU values of DAENet_4, DAENet_8, and DAENet_16 improved by 1.1%, 1.4%, and 0.7%, respectively. Their F1-scores improved by 1%, 1.2%, and 0.2%, respectively. In the Sentinel-1 test set, the mIoU values of DAENet_4, DAENet_8, and DAENet_16 improved by 1.9%, 0.4%, and 1.5%, respectively. Their F1-scores improved by 1.4%, 0.1%, and 1.1%, respectively. From the experimental results, the use of GP loss can effectively improve the accuracy of DAENet in oil spill detection.

### 3.4. Overall Ablation Study

Through the above experiments and the analysis and comparison of the experimental data, we conclude that the DAENet_8 network with Equation (12) as the joint loss function has the best accuracy and stability. In order to verify that both the DAEM and the new loss function are effective for model enhancement, a set of ablation experiments were conducted by combining different encoders and loss functions, and the experimental results are shown in Table 2.

The experimental results show that both the mIoU and F1-score metrics improved when using the new loss function or DAEM alone. Both the mIoU and F1-score metrics were the highest when the new loss function and DAEM were used simultaneously. The mIoU and F1-score were both improved by 2.3% for the PALSAR test set, while the mIoU and F1-score were improved by 2.6% and 1.6%, respectively, for the Sentinel-1 test set.

### 3.5. Validation Tests on the GaoFen-3 Dataset

**Figure 7:**
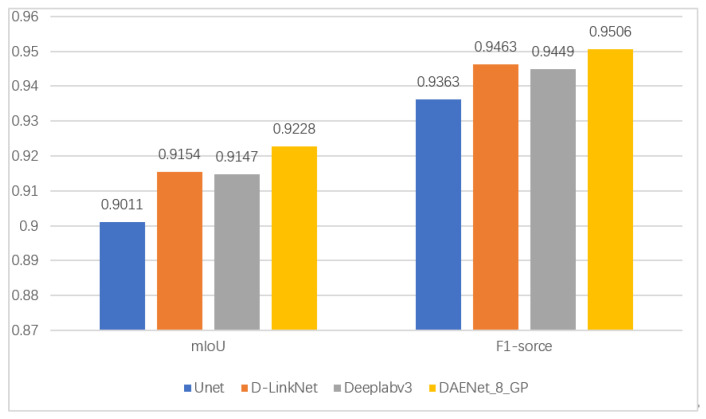
mIoU and F1-score of each model on the GaoFen-3 dataset.

To further validate the progress of DAENet using gradient profile loss in the SAR image oil spill segmentation task, we performed validation experiments on the GaoFen-3 dataset for the U-Net, D-LinkNet, DeepLabv3, and DAENet models. The model parameters were those used in the prior experiment above, and the experimental results are shown in Figure 7. The mIoU and F1-scores of DAENet were 92.28% and 95.06%, respectively. Compared with those for D_LinkNet, which was the highest scoring among the other models, the scores improved by 0.74% and 0.43%, respectively.

### 3.6. Implementation Details

In terms of details of the experimental process, the model proposed in this paper was implemented based on Python 3.9.12 (Guido van Rossum from Google) and PyTorch 1.11.0 (Facebook Artificial Intelligence Research Institute). We used the Adam optimizer with an initial learning rate of 2 × 10^−4^ and trained our network with a batch size of 16. At the same time, we used the learning rate optimization strategy. When the loss of three consecutive epochs was higher than the minimum loss, we adjusted the learning rate to 1/2 of the previous. When the learning rate was lower than 5 × 10^−7^, we discontinued learning. Sigmoid output was followed by values ranging from 0 to 1. We chose the default value of 0.5 as the threshold; prediction probabilities below the set threshold of 0.5 were judged as background, and those above the threshold of 0.5 were judged as oil spill targets. All models were trained on an NVIDIA RTX2080Ti 11GB GPU (NVIDIA, USA).

### 3.7. Visualization Result Analysis

**Figure 8:**
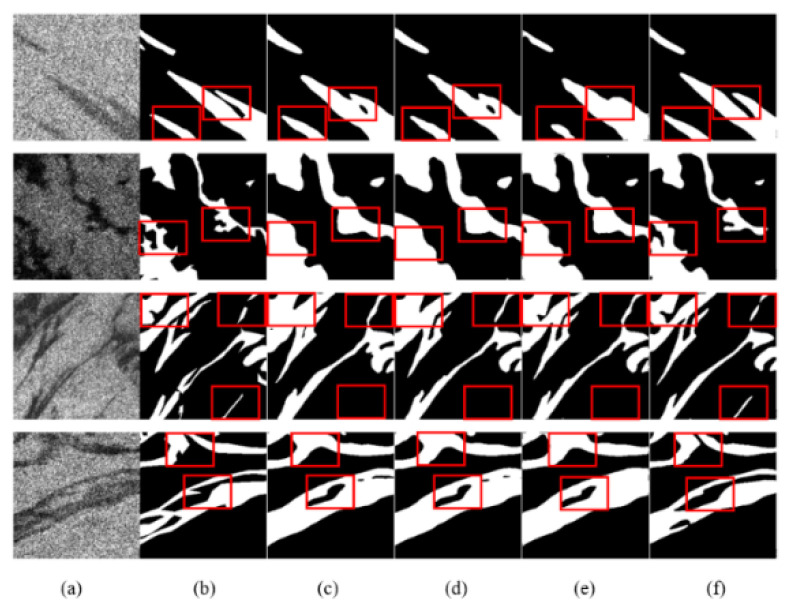
Qualitative comparisons with other methods on the PALSAR test set: (**a**) SAR image; (**b**) Ground truth; (**c**) U-Net; (**d**) D-LinkNet; (**e**) DeepLabv3; (**f**) DAENet.

**Figure 9:**
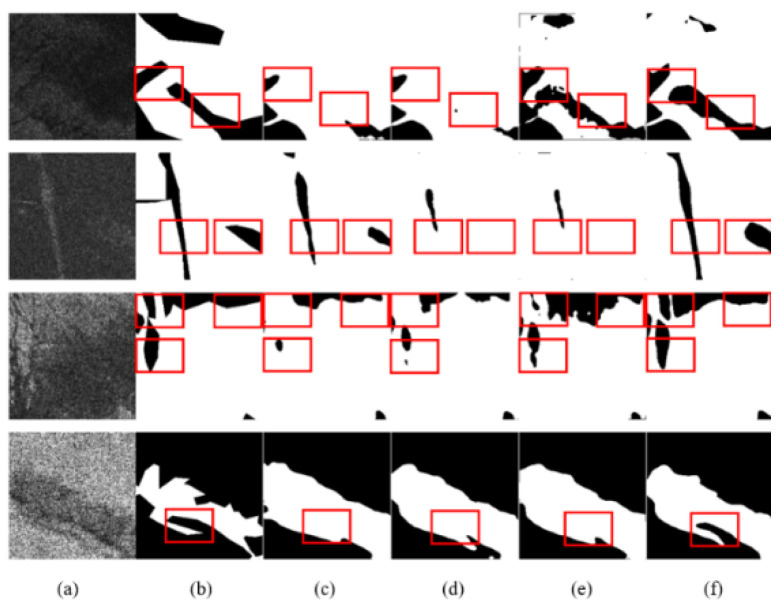
Qualitative comparisons with other methods on the Sentinel-1 test set: (**a**) SAR image; (**b**) Ground truth; (**c**) U-Net; (**d**) D-LinkNet; (**e**) DeepLabv3; (**f**) DAENet.

**Figure 10:**
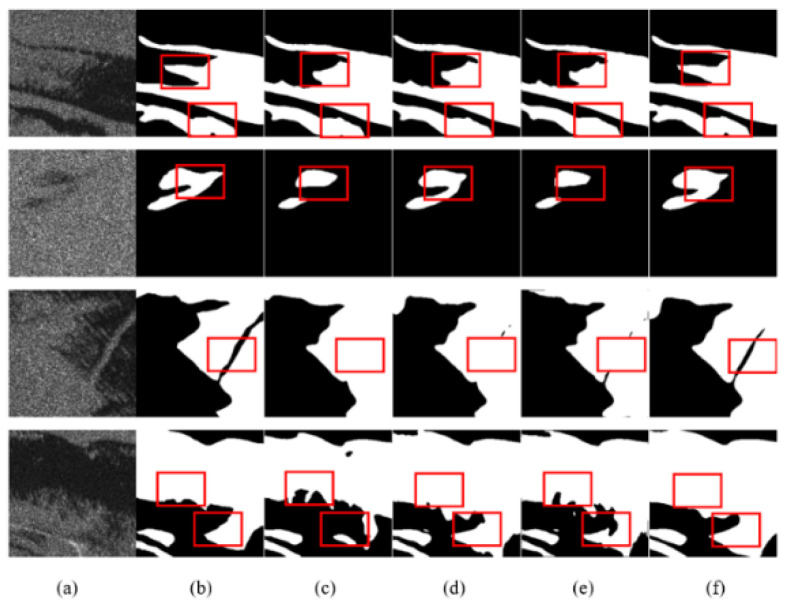
Qualitative comparisons with other methods on the GaoFen-3 dataset: (**a**) SAR image; (**b**) Ground truth; (**c**) U-Net; (**d**) D-LinkNet; (**e**) DeepLabv3; (**f**) DAENet.

The analysis of the visualization results showed that DAENet is superior to U-Net, D-LinkNet, and DeepLabv3 in the following aspects (Table 3): For oil films that exhibit a broken shape, DAENet can extract oil spill areas that are more consistent with the real situation, as shown in Figure 8 (third and fourth rows), Figure 9 (second row), and Figure 10 (third row). For images with small differences in gray values between the target and background regions, the DAENet model is less influenced and has a lower false positive rate, as can be seen in the first row of Figure 9. For some images with higher noise and more complicated edge conditions, the DAENet model can obtain more accurate extraction results of edge information, as seen in the first and second rows of Figure 8, the fourth row of Figure 9, and the first row of Figure 10. The red labels in the figure indicate false positive or false negative areas in the different model results.

It is worth noting that the test results on the GaoFen-3 dataset are better than those on the PALSAR dataset and the Sentinel-1 dataset in terms of both mIoU and F1-score, and there may be three main reasons for this phenomenon. First, by comparing the data labels in Figure 1 and Figure 3, it can be seen that the boundary of the oil spill area in the data labels in the GaoFen-3 dataset is smoother and closer to the real oil spill area, so the mIoU and F1-score of this dataset are higher in the Gaofen-3 dataset; further, there are fewer oil spill images with small differences in gray values between the background and target regions, so the false positive rate of the model is lower, which is supported by the experimental results. In the Gaofen-3 dataset, the difference between the test results of the DAENet model and those of the comparison models is lower compared to that for the other two test sets, and the DAENet model outperforms the comparison models in this respect. The reason for this situation is related to the spatial resolution of the original image, which is 12.5 m × 12.5 m in the PALSAR dataset. As shown in Table 4, the average mIoU and F1-score for different models in the PALSAR test results are 0.827 and 0.839, respectively. The spatial resolution of the images in the Sentinel-1 dataset is 5 m × 20 m, and the average mIoU and F1-score for different models in the corresponding test results are 0.838 and 0.885, respectively. The GaoFen-3 dataset has resolution 25 m × 25 m, and the average mIoU and F1-score for different models in the corresponding test results are 91.3% and 94.4%, respectively.

## 4. Conclusions

In this study, we proposed a dual attention encoding network (DAENet) for oil spill area recognition in SAR images. DAENet uses an encoder–decoder U-shaped structure to integrate multiscale features; global dependencies in spatial and channel dimensions were captured using the dual attention encoding module (DAEM). The gradient profile (GP) loss function explicitly takes into account spatial relationships over semantically constant regions by computing cosine similarities over horizontal and vertical spatial profiles in gradient space. The combined use of DAENet and GP loss effectively inhibited the effects of irregular shapes in SAR images and accurately obtained oil spill area segmentation results (mIoU 86.1%, F1-score 90.2% in the SOS dataset; mIoU 92.3%, F1-score 95.1% in the GaoFen-3 dataset). Although the recognition accuracy of the network algorithms proposed in this study is improved compared to that of the original model, the recognition accuracy still needs further improvement. In addition, different features of data must be analyzed and the advantages of different algorithms must be leveraged to obtain multiple features of data. In future work, different segmentation models and algorithms should be considered to obtain different features of data for the application of deep learning to improve marine oil spill detection using SAR images.

## Figures and Tables

**Table 1:** mIoU and F1-score of each model on PALSAR test set and Sentinel-1 test set.

	mIoU (PALSAR)	F1-Score (PALSAR)	mIoU (Sentinel-1)	F1-Score (Sentinel-1)
U-Net	0.818	0.831	0.832	0.882
D-LinkNet	0.827	0.838	0.831	0.882
DeepLabv3	0.829	0.839	0.838	0.881
DAENet_4	0.837	0.848	0.841	0.887
DAENet_8	0.836	0.849	**0.853**	**0.897**
DAENet_16	**0.841**	**0.851**	0.846	0.891

**Table 2:** Ablation experiments on different encoders and different loss functions, evaluated on the test SAR images in terms of the mIoU and F1-scores.

Row	Encoder	Loss Function	mIoU (PALSAR)	F1-Score (PALSAR)	mIoU (Sentinel-1)	F1-Score (Sentinel-1)
1	ResNet34	Dice + BCE + SSIM	0.827	0.838	0.831	0.882
2	ResNet34	Dice + BCE + GP	0.833	0.843	0.842	0.887
3	DAEM	Dice + BCE + SSIM	0.836	0.849	0.853	0.897
4	DAEM	Dice + BCE + GP	**0.850**	**0.861**	**0.857**	**0.898**

**Table 3:** Example of advantages in the visualization results.

Advantage	Example
Broken shape areas	Figure 8, third row, fourth row; Figure 9, second row; Figure 10, third row
Small differences in gray values between the target and background areas	Figure 9, first row
Higher noise and more complicated edge areas	Figure 8, first row, second row; Figure 9, fourth row; Figure 10, first row

**Table 4:** mIoU and F1-score comparison of test sets with different spatial resolutions.

Test Set	Spatial Resolution	Average mIoU	Average F1-Score
PALSAR	12.5 m × 12.5 m	0.827	0.839
Sentinel-1	5 m × 20 m	0.838	0.885
GaoFen-3	25 m × 25 m	0.913	0.944

## Data Availability

SOS data are available from http://grzy.cug.edu.cn/zhuqiqi/en/yjgk/32384/list/index.htm (accessed on 18 March 2022).
